# Medical Students’ Clinical Knowledge of Integrated Management of Childhood Illness: A cross-sectional comparative study

**DOI:** 10.18295/squmj.1.2024.006

**Published:** 2024-05-27

**Authors:** Zamzam Al Abri, Maisa Al Kiyumi, Sanjay Jaju, Muna Al Saadoon

**Affiliations:** 1 College of Medicine and Health Sciences, Sultan Qaboos University, Muscat, Oman; 2 Department of Family Medicine and Public Health, Sultan Qaboos University Hospital, University Medical City, Muscat, Oman; 3 Department of Family Medicine & Public Health; 4 Department of Child Health

**Keywords:** Pediatrics, Disease Management, Students, Undergraduate Medical Education, Oman

## Abstract

**Objectives:**

This study aimed to investigate and compare the clinical knowledge implications of the integrated management of childhood illness (IMCI) preservice education between pre-clerkship and junior clerkship medical students.

**Methods:**

This observational comparative cross-sectional study was conducted between June and August 2022 at Sultan Qaboos University, Muscat, Oman. A self-administered questionnaire was utilised and included questions on sociodemographic data, duration of IMCI preservice training, knowledge of the participants concerning the IMCI objectives and information on a range of childhood conditions.

**Results:**

A total of 97 medical students were included in the study. The majority of students (42.3%) had received 2 lectures in IMCI preservice training. The role of the IMCI approach in reducing childhood morbidity and mortality was advocated by the majority of students (80.8% in the junior-clerkship [JCR] group and 73.3% in the pre-clerkship group). The awareness of the IMCI component of improving the health system was higher in JCR compared to pre-clerkship participants (*P* = 0.044). When compared to pre-clerkship students, the JCR participants demonstrated a slightly higher awareness of skin pinch (*P* = 0.038), chest indrawing (*P* = 0.008), anaemia assessment based on nail bed examination (*P* = 0.002), diagnostic assessment of malnutrition based on palm examination (*P* = 0.018), sucking capacity in breastfeeding (*P* = 0.025), and vaccines such as those for tuberculosis (*P* = 0.001), pneumococcal (*P* = 0.018) and rotavirus (*P* = 0.007).

**Conclusion:**

The majority of students displayed good IMCI knowledge and JCR students showed better knowledge compared to pre-clerkship candidates.

**Advances in Knowledge**

- *This study was the first conducted in Oman to evaluate the influence of integrated management of childhood illness (IMCI) pre-service education on the clinical knowledge of medical students.*

**Application to Patient Care**

- *Enhancing pre-service education in IMCI for undergraduate medical students will lead to improved patient care by strengthening their clinical knowledge and skills applied in practice.*

Infectious conditions disproportionately influence the health and wellness of underprivileged paediatric populations across the globe.^1^ Accordingly, the World Health Organization (WHO) initiated a project to maintain a database of childhood diseases, including malaria, pneumonia and diarrhea based on their prevalence in children aged below 5 years.^2^ The findings from the contemporary literature revealed high preventable mortality rates in children below 5 years (5.2 million children), which can be attributed to the increasing prevalence of infectious conditions, such as malaria, diarrhea and pneumonia.^3^ Based on a recent analysis from 2020, diarrhea affected 1.7 billion children worldwide, resulting in the deaths of 5.25 × 10^5^ children.^4^ The prevalence rate of malaria in children is 35.4%, and 151 million new malaria cases are reported across the globe annually.^5^^,^^6^ Oman is currently experiencing a widespread outbreak of childhood diseases such as diarrhea.^7^ A decade ago, a large number of malaria cases were reported in Oman, but diarrhea was even more prevalent (~20%).^7^^,^^8^

The integrated management of childhood illness (IMCI) is an innovative approach devised by the WHO to counter the high incidence of childhood diseases and their deleterious outcomes.^9^ The primary aim of the IMCI intervention is to enhance comprehensive medical training and education among healthcare professionals to foster the overall health and wellness of paediatric populations. It also aims to enhance the nutrition and feeding practices in children to safeguard their health and reduce the occurrence of preventable illnesses.^10^ The IMCI strategy promotes the precise diagnosis of childhood illnesses in outpatient settings within health facilities. It also ensures the appropriate and comprehensive treatment of all significant conditions affecting young children. Additionally, it strengthens caregiver counselling and expedites the referral process for severely ill newborns and children. In household settings, it encourages appropriate care-seeking behaviours, improved nutrition and support for early childhood development, prevention of illnesses and the correct implementation of prescribed care and adherence to treatment.^9^ Notably, the IMCI approach consists of 3 main components: (1) improving the case management skills of healthcare providers; (2) reinforcing health systems to offer high-quality care; and (3) enhancing family and community health practices to promote overall health, growth and development.^9^

A cross-sectional survey conducted in 95 countries revealed that the IMCI strategy was fully implemented in at least 90% of their districts.^11^ Notably, nations that fully implemented the IMCI strategy were 3.6 times more likely to achieve the Millennium Development Goal 4 compared to countries that did not.^11^

The WHO recommends the implementation of IMCI training sessions for undergraduate students due to its potential role in improving their clinical knowledge.^12^ The results from a recent cross-sectional study indicated the advantage of the IMCI approach in improving the case management and disease assessment expertise of undergraduate medical students.^13^ These outcomes warrant the enhancement of medical education to effectively improve the clinical skills of medical students in concordance with the evolving healthcare demands of paediatric populations. The IMCI was formally incorporated into Oman’s healthcare system in 1999. In 2007, the IMCI was incorporated into the medical degree (MD) programme curriculum at Sultan Qaboos University (SQU). The MD programme at SQU is structured into three distinct phases. Phase 1, which commences in the first year, concentrates on imparting foundational knowledge. Phase II spans the second and third years, offering a comprehensive academic experience. Phase IIIA, starting at the beginning of the fourth year, is dedicated to the pre-clerkship phase, emphasising clinical readiness. Finally, phase IIIB encompasses both junior and senior clerkships, extending through the fifth and sixth years, respectively, providing students with extensive practical experience in clinical settings. These well-defined phases provide students with a guided path as they progress through their medical education. The IMCI was integrated into lectures of 1-hour duration during phase II. These lectures aimed to introduce students to the IMCI, its components, common childhood disorders and treatment concepts. In phase III, students get additional opportunities to practice the IMCI strategy at primary healthcare facilities. In addition, the IMCI was incorporated into the rotations’ summative assessments.

Initiating IMCI preservice education during the early stages of medical school (phase II) may be challenging due to students’ limited exposure to clinical practice. The primary challenge to the integration of IMCI into the MD programme persists due to the absence of solid evidence substantiating its implementation, despite its incorporation into the MD programme curriculum more than 15 years ago. Therefore, this study aimed to determine the impact of IMCI pre-service education on pre-clerkship and junior-clerkship (JCR) medical students’ clinical knowledge at SQU, Muscat, Oman.

## Methods

This comparative cross-sectional study was conducted between June and August 2022 at the College of Medicine and Health Sciences at SQU. The inclusion criteria were undergraduate medical students in phase III (IIIA: pre-clerkship and IIIB: junior-clerkship) who had attended at least 1 session on IMCI. Exclusion criteria were students who had never attended any IMCI sessions and those who declined to participate. Convenient sampling was used for recruitment. Eligible students were invited to participate, and the purpose of the study was explained. The enrolled participants were assigned a unique number in chronological order.

The eligible participants were invited to fill in a self-administered paper-based semi-structured questionnaire using a convenient sampling method. The questionnaire consisted of 2 parts. Part 1 included the sociodemographic characteristics, such as age, gender, educational level and duration of pre-service IMCI training received. Part 2 consisted of 10 IMCI case-based close-ended questions addressing their clinical knowledge of the IMCI objectives and components. Objectives include reducing morbidity and mortality of <5-year-old children and promoting the growth and development of children by counselling the mothers and caretakers. Components include improving case management skills of health workers, improving the health system and improving family and community practice. The questionnaire further included information on a range of childhood conditions, including anaemia, pneumonia and diarrhea, and their attributes (e.g. symptomatology) and disease prevention (e.g. vaccination) [Supplementary Tables 1 and 2]. Permission to use the questionnaire was obtained from the corresponding author.^14^

The sample size was determined for a finite population in phases IIIA and IIIB (118 and 128 students, respectively), using the Krejcie and Morgan table.^15^ A sample size of 95 students was required. Investigators utilised a convenient sampling approach to recruit the study subjects.

Data were analysed using Statistical Package for Social Sciences (SPSS), Version 26 (IBM Corp., Armonk, New York, USA). The sociodemographic characteristics and results were displayed as descriptive statistics in frequency tables and bar charts. The responses between the participant groups and relevant variable categories were tested for statistical significance by the Chi-squared test of association. A *P* value of less than 0.05 was considered statistically significant.

Informed written consent was obtained from all individuals who agreed to participate. Furthermore, the process was anonymous to maintain the confidentiality of the students. Ethical approval was obtained from the Medical Research Ethics Committee, College of Medicine and Health Sciences, SQU (MREC #2757).

## Results

A total of 97 medical students participated in this study. The respondents were between the ages of 20–24 years. The majority of the respondents were females (n = 60, 61.9%), and a smaller number were males (n = 37, 38.1%). The numbers of JCR and pre-clerkship students were 52 and 45, respectively. Remarkable variations in the number of IMCI training sessions were observed among the participants; 28.9% of respondents received 1 lecture or tutorial, 42.3% received 2 lectures or tutorials, 1.0% received 3 lectures or more and 27.8% received practical sessions at family medicine rotation [Table 1].

The analysis of the first IMCI objective revealed the agreement of 73.3% of pre-clerkship and 80.8% of JCR participants regarding the role of IMCI training sessions in reducing morbidity and mortality in paediatric populations. The second IMCI objective, which is the role of caregiver counselling in improving child growth, was advocated by 57.8% and 61.5% of pre-clerkship and JCR students, respectively. However, nearly one-third of students had no insight into their knowledge levels concerning the caregiver counselling implications in the developmental outcomes of the paediatric populations [Table 2].

**Figure 1:**
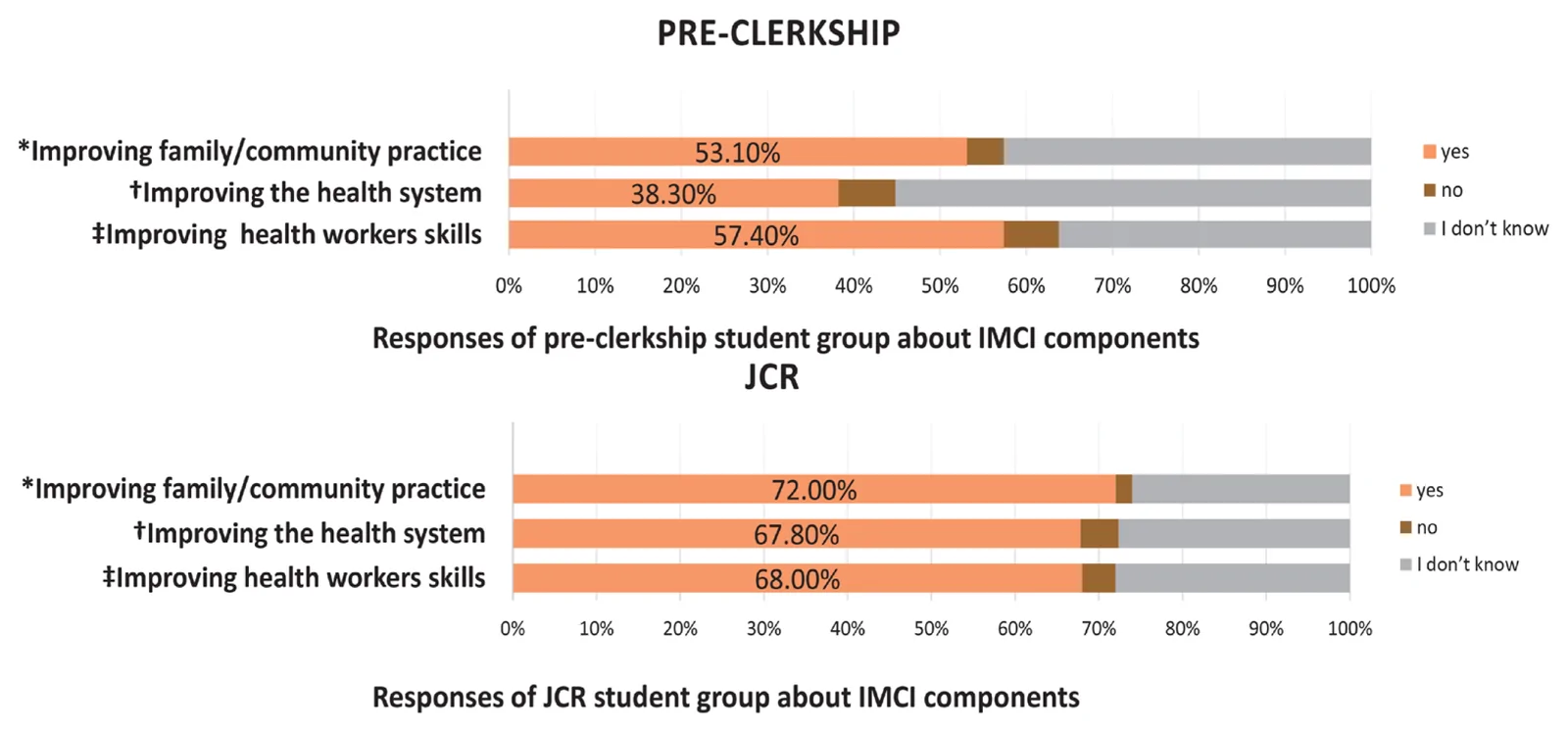
Responses of medical student groups regarding integrated management of childhood illness components. *IMCI = integrated management of childhood illness; JCR = junior-clerkship*. *P = 0.18 †P = 0.044 ‡P = 0.588

The responses from 57.4% of pre-clerkship and 68.0% of JCR students indicated the influence of the first IMCI component on the skill improvement of healthcare workers. In addition, 38.3% of pre-clerkship and 68.0% of JCR students advocated the healthcare system improvement implications of the IMCI training sessions. However, 53.1% and 74.9% of the pre-clerkship and JCR students, respectively, indicated the community and family practice benefits of the IMCI training approach [Figure 1].

Questions 3–8 were designed to investigate the clinical features, examination and diagnostic implications of the IMCI training strategy. They particularly evaluated the clinical practice applications of the IMCI approach in the assessment of severe dehydration, malnutrition, anaemia, pneumonia and diarrhea. The findings revealed the advocacy of 66.0% and 78.0% of pre-clerkship and JCR participants regarding the significance of assessing the consciousness levels of paediatric patients with diarrhea. Most participants (89.4% of pre-clerkship and 80.0% of JCR) provided the consensus to examine the general condition of diarrhea-affected children to improve their disease management effectively. The findings revealed that the students possessed knowledge of skin pinch. Nevertheless, when compared to pre-clerkship students, JCR participants demonstrated a slightly higher awareness of this diagnostic assessment (82.0% versus 66.0%, *P* = 0.038) [Table 3].

Except for the breathlessness attribute, most of the JCR students were aware of the clinical features of pneumonia. However, 46.8% of pre-clerkship students were unaware of the significance of chest in-drawing, while 42.6% of them knew this diagnostic attribute. Notably, a significant difference was observed in the knowledge of the clinical manifestation of pneumonia related to chest in-drawing between pre-clerkship and JCR students (*P* = 0.008).

**Figure 2:**
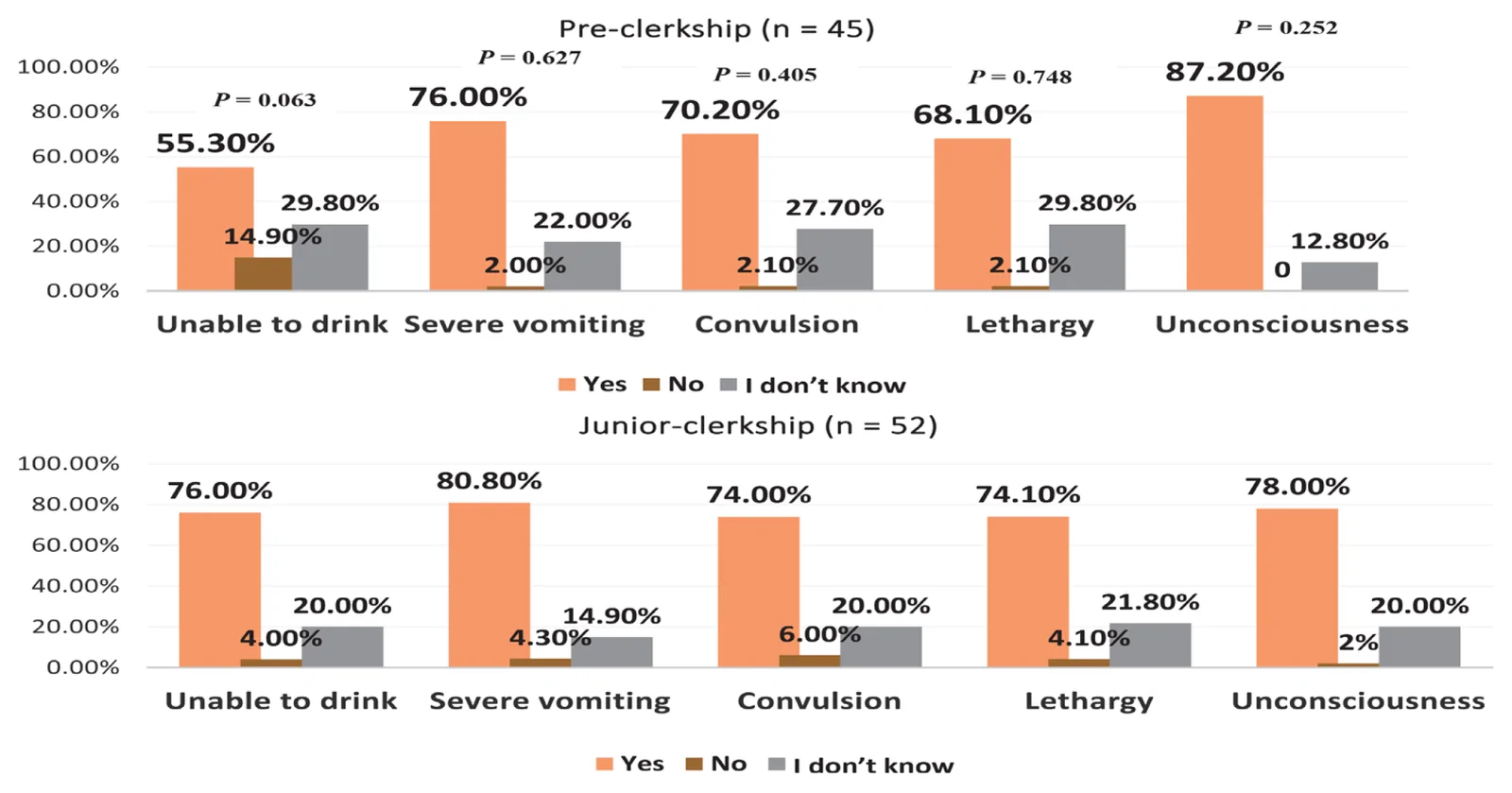
Responses of student groups regarding the clinical signs of paediatric conditions. *JCR = junior-clerkship*.

The JCR students were more aware of the clinical signs or manifestations of anaemia. A total of 40.4% of pre-clerkship students were aware of the nail bed examination, while a higher percentage of the JCR students had awareness of the tongue, palm and conjunctive assessments. Compared to the pre-clerkship students, JCR participants had better knowledge and understanding of the diagnostic assessment of malnutrition, based on the systematic evaluations of growth factor charts, palms (*P* = 0.018) and ankle edema. The unconscious sign of dehydration was better understood by 82.4% of JCR students, compared to 72.3% of those with pre-clerkship enrolment. In comparison with the JCR participants, pre-clerkship students had a better insight into the assessment of skin tone loss, sunken eyes and latency. A total of 80.9% of the pre-clerkship candidates had better knowledge of the general condition of infants compared to 78.4% of the JCR participants; 80.4% of the JCR students had a better insight into the sucking capacity of infants/babies compared to 72.3% of the pre-clerkship participants (*P* = 0.025). The findings further revealed better knowledge among JCR students, concerning the characteristics of vaccine-preventable diseases in paediatric populations. For example, 78.4% of JCR students knew the pneumococcal vaccine, compared to 45.7% of pre-clerkship students (*P* = 0.008). However, polio and measles-related awareness was found to be higher in pre-clerkship students compared to the JCR candidates though the difference was not statistically significant (*P* = 0.473 and *P* = 0.568, respectively). Similarly, a greater number of JCR students understood the danger signs of paediatric conditions, including unconsciousness, lethargy, convulsions and vomiting [Figure 2].

## Discussion

The overall results of this cross-sectional study indicated good IMCI knowledge among most participants and better IMCI knowledge among JCR respondents compared to the pre-clerkship candidates. The higher engagement of the JCR students in the preservice IMCI training sessions, as well as the impact of seniority and greater clinical exposure in paediatric rotations, was the probable cause of their enhanced childhood disease management knowledge compared to those with pre-clerkship enrolment. The study findings revealed knowledge of IMCI components and objectives among most participants. However, this finding is contrary to the results of Khatun *et al*., which indicated IMCI awareness among only half of their respondents.^14^

Approximately 60% of the participants in the present study had a thorough knowledge of paediatric diarrhea management while Joshi *et al*. found a lack of awareness of diarrhea assessment in 55.5% of study subjects.^16^ The present study findings further revealed a thorough knowledge of the clinical attributes of paediatric pneumonia in the majority of JCR participants, comparing favourably with the pre-clerkship candidates. In addition, most students in this study had a sound knowledge of anaemia assessment, in contrast to the findings of a study by Khatun *et al*.^14^

Most students in this study were found to have a concrete understanding of paediatric malnutrition-related growth charts. This outcome was contrary to the results of a prospective single-arm study by Sahu *et al*. indicating a lack of growth chart assessment and monitoring in Puducherry, India.^17^ The present study further revealed the knowledge of two-thirds of students concerning the malnutrition assessment of 2-month-old infants via their ankle edema assessment. This finding challenged the outcomes of Joshi *et al*. which revealed a lack of malnutrition assessment knowledge among respondents.^16^ Importantly, most students in the current study were found to be aware of severe dehydration manifestations, including loss of skin tone, dilated eyes, latency in memory responses and unconsciousness. These findings concurred with the outcomes of Shaheen *et al*. indicating the knowledge of water intake recommendations, dehydration prevention and dehydration definition among the majority of participants.^18^

The findings of the current study indicated insignificant differences regarding the knowledge of vaccine-preventable diseases between the two groups. The overall results revealed a thorough knowledge among most participants regarding the role of vaccination in preventing various disease conditions. The current study’s results are in-line with Khatun *et al*.’s study concerning the awareness of vaccine-preventable diseases, excluding mumps, among participants.^14^ The results also supported the concept of improving vaccination knowledge among medical students to reduce the incidence of communicable diseases in paediatric populations.^19^^,^^20^

The findings of this study revealed a generally good comprehension of the clinical knowledge implications of preservice IMCI training among undergraduate medical students. These results underscore the importance of enhancing the IMCI knowledge base of medical students to effectively manage infant morbidity and mortality rates, thereby promoting the overall growth and development of paediatric populations. As this is the first study conducted in Oman about the influence of IMCI pre-service training on the knowledge of medical students, it is anticipated that the current findings will contribute valuable data to the existing literature. Future research should employ qualitative group discussions to acquire data regarding medical students’ knowledge of IMCI preservice training. These group discussions will assist in minimising ambiguity in responses and adding clarity to the final results.

The primary limitation of the current study lies in its cross-sectional design, which hindered the establishment of a causal relationship. In addition, the small sample size further restricted the generalisability of the analysis. The results of this study are also not devoid of possible recall bias based on the self-administered questionnaire. Importantly, the studies focusing on IMCI were limited, and the variability in the duration of IMCI training across different studies made direct comparisons with the findings of the current study challenging. Finally, the absence of a pre-post-intervention comparison could have influenced the study findings.

## Conclusion

The IMCI approach is a holistic strategy to diagnose and manage a range of simple-to-complex childhood illnesses efficiently. Results from this study indicated an overall good understanding of the preservice IMCI training’s clinical practice implications among undergraduate medical students. The IMCI-related knowledge of the JCR student group was found to be stronger than that of the pre-clerkship students, probably due to their higher engagement in the IMCI preservice training and family medicine and paediatric rotations. These results advocate the need for improving the IMCI knowledge base of medical students to control infant morbidity and mortality rates effectively while improving the overall growth and development of paediatric populations. Future qualitative studies should evaluate these findings with wider student populations.

## Supplementary Information



## Figures and Tables

**Table 1:** Characteristics of participating pre-clerkship and junior clerkship medical students from Sultan Qaboos University, Muscat, Oman (N = 97)

Characteristic	n (%)
**Age in years**	
20	1 (1)
21	12 (12.4)
22	41 (42.3)
23	36 (37.1)
24	7 (7.2)
**Gender**	
Male	37 (38.1)
Female	60 (61.9)
**Educational level**	
Phase IIIA pre-clerkship	45 (46.4)
Phase IIIB JCR	52 (53.6)
**Pre-service IMCI training duration**	
1 lecture/tutorial	28 (28.9)
2 lectures/tutorials	41 (42.3)
≥3 lectures/tutorials	1 (1)
Practical sessions at the JCR family medicine rotation	27 (27.8)

JCR = junior-clerkship; IMCI = integrated management of childhood illness.

**Table 2:** Medical student groups’ responses based on their knowledge of the integrated management of childhood illness’ first and second objectives

Group	n (%)*			*P* value	n (%)†			*P* value
	Students with knowledge of the IMCI’s first objective	Students with no knowledge of the IMCI’s first objective	Students without any awareness of their knowledge of the IMCI’s first objective		Students with knowledge of the IMCI’s second objective	Students with no knowledge of the IMCI’s second objective	Students without any awareness of their knowledge of the IMCI’s second objective	
**Pre-clerkship (n = 45)**	33 (73.3)	2 (4.4)	10 (22.2)	0.624	26 (57.8)	2 (4.4)	17 (37.8)	0.745
**JCR (n = 52)**	42 (80.8)	1 (1.9)	9 (17.3)		32 (61.5)	4 (7.7)	16 (30.8)	

IMCI = integrated management of childhood illness; JCR = junior-clerkship.

* First objective = to reduce morbidity and mortality of children under the age of 5 years.

† Second objective = to promote the growth and development of children by counselling the mothers and caretakers.

**Table 3:** Responses of medical student groups concerning the diagnostic assessment of diarrhea, pneumonia, anaemia, malnutrition, severe dehydration, difficulty in breastfeeding and vaccine-preventable diseases

Characteristic	Response, %						*P* value
	Pre-clerkship			JCR			
	Yes	No	Don’t know	Yes	No	Don’t know	
**Examination of diarrhea**							
Level of consciousness	66.0	6.4	27.7	78.0	2.0	20.0	0.228
General condition of the body	89.4	0.0	10.6	80.0	0.0	20.0	0.159
Skin pinch	66.0	10.6	23.4	82.0	0.0	18.0	0.038
Eye examination	61.7	6.4	31.9	68.0	8.0	24.0	0.532
**Clinical features of pneumonia**							
Breathlessness	78.7	6.4	14.9	78.0	0.0	22.0	0.144
Cough	83.0	4.2	12.8	84.2	2.2	13.6	0.389
Increased respiratory rate	76.6	2.1	21.3	80.0	4.0	16.0	0.556
In-drawing of chest	42.6	10.6	46.8	68.0	0.0	32.0	0.008
**Anaemia assessment**							
Examination of the conjunctiva	76.6	6.4	17.0	82.0	0.0	18.0	0.163
Examination of the nail bed	40.4	31.9	27.7	66.0	6.0	28.0	0.002
Examination of the palm hand	59.6	8.5	31.9	72.0	2.0	26.0	0.238
Examination of the tongue	40.4	19.1	40.5	48.2	7.8	44.0	0.204
**Paediatric assessment for malnutrition**							
Examination of ankle edema	53.2	17.0	29.8	62.4	7.3	29.3	0.603
Examination of the palm	25.5	34.1	40.4	52.0	16.0	32.0	0.018
Assessment of the degree of malnutrition for the growth chart	93.6	0.0	6.4	86.0	0.0	14.0	0.331
**Signs of severe dehydration**							
Unconsciousness in child	72.3	8.5	19.2	82.4	0.0	17.7	0.264
Latency	80.9	2.1	17.0	76.5	0.0	21.5	0.914
Sunken eyes	91.5	0.0	8.5	78.4	2.0	19.6	0.214
Loss of skin tone	68.1	10.6	21.3	62.7	11.8	25.5	0.942
**Examination of difficulty in breastfeeding**							
The general condition of the baby	80.9	0.0	19.1	78.4	2.0	19.6	0.580
Sucking capacity	72.3	12.8	14.9	80.4	0.0	19.6	0.025
Physical attachment of baby with mother	57.4	6.4	36.2	70.6	2.0	27.5	0.341
**Vaccine-preventable diseases**							
Tuberculosis	45.7	26.1	28.3	80.4	3.9	15.7	0.001
Polio	87.0	2.2	10.9	84.3	0.0	15.7	0.473
Measles	89.1	0.0	10.9	80.4	2.2	17.4	0.568
Diphtheria	73.9	2.2	23.9	82.4	0.0	17.6	0.364
Whooping cough	80.4	2.2	17.4	85.4	2.0	12.6	0.554
Tetanus	69.6	6.5	23.9	84.3	0.0	15.7	0.072
Hepatitis B	76.1	8.7	15.2	80.0	2.8	17.2	0.301
Mumps	84.8	0.0	15.2	87.0	2.2	10.9	1.000
Rubella	80.4	2.2	17.4	80.9	0.0	19.1	0.522
Pneumococcal (conjugate)	45.7	10.9	43.4	78.4	3.9	17.6	0.008
Haemophilus influenza type B	63.0	10.9	25.1	80.4	3.9	15.7	0.111
Rotavirus	37.0	8.7	54.3	58.8	17.6	23.5	0.007

JCR = junior-clerkship.
